# RootHairAreaFinder: an image processing method for quantifying barley root growth and root hairs simultaneously in a flat rhizotron system

**DOI:** 10.1186/s13007-026-01564-z

**Published:** 2026-07-12

**Authors:** George Themistokleous, Kirsty Binnie, Blair M. McKenzie, Sacha J. Mooney, Tracy A. Valentine

**Affiliations:** 1 Ecological Sciences, The James Hutton Institute, Invergowrie, Dundee, DD2 5DA Scotland, UK; 2 Division of Agricultural & Environmental Sciences, University of Nottingham, Sutton Bonington Campus, Sutton Bonington, Leicestershire LE12 5RD UK; 3 Eratosthenes Centre of Excellence, Lemesos, Cyprus; 4 Geography and Environmental Science, School of Social Sciences, University of Dundee, Nethergate, Dundee, DD1 4NH Scotland, UK

**Keywords:** Root hairs, Soil physics, Image analysis, Rhizotron, Dry bulk density, Drought

## Abstract

Root system architecture and root hairs highly influence plant resource uptake, yet their simultaneous quantification at the whole-plant scale remains challenging due to the conflicting requirements of high-resolution imaging and non-destructive, repeated measurements. Here, we present a plant growth and imaging system based on A3 sized flatbed rhizotrons, combined with a non-machine-learning image analysis workflow implemented in R, that enables repeated, in situ quantification of barley root system architecture and accompanied visible projected root hair area (VP root hair area) over three weeks of growth. The system produces integrated outputs highlighting both whole-root architecture and the spatial distribution of surrounding root hairs from single images. Validation of the image analysis algorithm showed good segmentation performance, with average Matthews Correlation Coefficient values of 0.68 for projected root area and 0.65 for VP root hair area. To demonstrate its experimental applicability, the system was used to assess root growth and root hair responses under controlled environmental conditions, combining three irrigation regimes (2, 4, and 6 irrigation events per day) with three dry bulk density levels (1.4, 1.5, and 1.6 g cm^−3^). In addition to whole-system metrics, the approach enables analysis of root hair expansion at individual root tips. This methodology provides a training-free method for integrated analysis of root traits e.g. projected root size, root growth, root diameter and root hairs under controlled physical conditions similar to soil, facilitating studies of root–soil interactions that require both spatial resolution and temporal continuity.

**Supplementary Information:**

The online version contains supplementary material available at https://doi.org/10.1186/s13007-026-01564-z.

## Background

Global food security is a major challenge for current and future generations [1]. Major issues such as drought stress negatively influence plant growth, survival and reproduction [2] and are responsible for large reductions in crop yield [3]. Drought severity is also projected to increase due to increasing global temperatures [4]. The projected increase in average global temperature [5] means that roots will experience a higher frequency of mechanical impedance, as reduction in water content of soil is often correlated to an increase in soil strength [6, 7]. Compaction and climate change can also increase the risk of flooding events due to reduction in pore size distribution of the soil and the increase in heavy rainfall events. The above climatic effects can be exacerbated when one considers the negative consequences on soil compaction brought upon by heavy agricultural machinery [8]. As such, it is important to understand how the soil physical conditions of water and mechanical stresses interact to impact root growth [9, 10].

A potential mechanism for overcoming the negative effects associated with drought are changes in root system architecture (RSA), which are of immense importance for the survival and growth of the plant [11, 12]. Changes in RSA that mitigate drought stress can involve deep rooting, as this enables the uptake of water from deeper soil layers [13]. In addition, higher root density, increased lateral root production, and alterations in root diameter can improve water extraction efficiency, enhance soil penetration, and increase the capacity of plants to exploit heterogeneous water resources within the soil profile [14, 15].

Root hairs have also been considered to aid plants in overcoming drought effects [16]. They are a common anatomical characteristic of most vascular plants which can dramatically increase the surface area of roots [17]. They influence the uptake of both nutrients and water [18] with the root hair zone being the most active zone for fluid transfer in the root system due to its high permeability [19]. In more precise terms, they are extensions of trichoblasts, specific root epidermal cells which develop in the maturation zone of the root tip [20]. Root hair phenotypes can be dynamic, and drought conditions can induce increases in root hair length, thereby expanding the total root surface area and enhancing water absorption capacity [21]. Furthermore, root hairs can improve soil penetration and root-soil contact [22], particularly in compact soils [23], where increased soil compaction can reduce root hair length [24].

However, unlike other easily accessible plant organs such as the stem and leaves or small embryo seeds, the imaging of undisturbed root systems is a more complicated process due to the opacity of soil. Recent advances in imaging enable in situ quantification of root hairs grown in soil by using synchrotron imaging [25, 26], however, the reported dimensions for this plant-soil microcosm are currently small as the germinated seedlings were grown inside syringes with a diameter of only 3 cm. An alternative and less complex method for assessing root growth in situ are rhizotrons. Rhizotrons enable non-destructive imaging of root systems grown in closed containers filled with a growth substrate [27–29]. In addition to coarse root system structures, i.e., roots, it is also possible to image and quantify finer features of the root system such as root hairs [30].

Root hair analysis methods are available for excised roots, for example Root Hair Sizer [31] and DIRT/µ [32], however, automated root detection in rhizotron systems is a rather complicated process, because of the complex root background with image quality varying with soil conditions, lighting and root colour and as such, unsupervised machine learning methods have mixed performance results [33]. As a result, most of the available software require the user to manually perform the root segmentation from the background [34]. In a similar manner, Vincent et al. [30] developed a semi-automated method for analysing root soil images obtained from rhizotron tube cameras using training images. More recently, Lu et al. [35] used a machine learning (ML) based U-net model and transfer learning to segment root hairs with very high accuracy. However, ML methods require prior model calibration for optimal performance. In addition, if tube based rhizotron are used each imaging apparatus (1.1 × 1.1 × 1.2 cm^3^) installed only captures spatially localized root information, resulting in numerous small images. As such, it is desirable to develop easy to use, non-computationally demanding, and sufficiently accurate algorithms to measure basic root hair properties that are spatially linked in situ, under realistic growth conditions (for example the characterisation of root hairs linked to their spatial position in the root system).

In this paper, a flatbed A3 sized rhizotron system (RS) is introduced along with an image processing algorithm which enables the detection and quantification of the visible root system and its visible projected root hair area (VP root hair area) for barley seedlings grown for up to a period of three weeks. The algorithm is fully automated and can be run from the R console [36] and, is designed for time-lapse studies that investigate root and root hair development. The proposed method is then demonstrated in a phenotyping experiment involving spring barley (*Hordeum vulgare* var. Optic) subjected to different sand water concentrations and dry bulk densities.

## Methods

### Plant growth system and imaging

Seeds of *Hordeum vulgare* c (Optic) were obtained from the collections at the James Hutton Institute, Dundee, UK and had been previously stored at 4 °C. Plants were grown in a rhizotron system, as described in Supplementary file 1. Briefly, the rhizotron system was constructed of Perspex acrylic sheets approximately A3 size (420 height × 15 depth × 297 width mm^3^), with side spacer bars, and an automatic irrigation system. Rhizotrons were filled with a dry, black-coloured sand mix from Seachem (Flourite Black). Details about its physical properties, including penetrometer resistance [37] and volumetric water loss across a range of matric potentials, are provided in Supplementary file 2. A controlled release fertiliser, i.e., “Scotts Osmocote”, manufactured by “Everris” was included by individually grinding to dust 0.3 g of fertilizer with a mortar and pestle for each rhizotron and subsequently mixing the fine powder thoroughly with the required mass of rhizotron substrate prior to rhizotron packing. Images were obtained using an A3 sized flatbed scanner (EPSON Expression 12000XL) at 1,200 dpi, on days 7, 14 and 21 post transfer of seedlings to the rhizotrons.

Water content was adjusted using an automated irrigation system. After 3 days of water equilibration, pre-germinated seeds, germinated as described in Valentine et al. [38], were transferred to the rhizotrons. Each rhizotron was covered in black sheeting (Do4U) to exclude light, placed at an angle of 45° to the horizontal and kept in a glasshouse with the temperature set to 18 °C during the day and 14 °C during the night. Artificial lighting was activated at 150 W m^−2^ from GAVITA GAN 400AL Ecomax lamps fitted with 400W SON-T sodium bulbs.

To analyse the effect of physical conditions on root hair distribution, a two-way 3 × 3 ANOVA experiment was used with the factors being dry bulk density (DBD) and number of irrigation events (IRE), i.e., water concentration. The experiment had a randomized (blocked) structure with the RS units arranged in two parallel linear rows with 4 treatment replicates. The treatments for DBD were 1.4, 1.5 and 1.6 g cm^−3^ and the treatments of IRE were 2, 4 and 6 irrigation events per day with an event having a duration of 3 min. The amount of water added was adjusted to maintain an approximate degree of saturation for each treatment.

### Assessment of image quality

Prior to the selection of the 2 mm thick acrylic, the effect of different imaging surfaces on image quality was first assessed. Three surfaces were considered: (1) 2 mm thick acrylic, (2) 4 mm thick, low iron, reinforced glass, and (3) 5 mm thick acrylic. To objectively assess image quality, an image sharpness index was used. The Image Sharpness Index (ISI) is a dimensionless measure of image sharpness, which is computed by estimating the average value of the local greyscale variance at the scale of interest [39]. The scale selected was a block radius of 1 pixel, because root hairs are only 1 or 2 pixels thick at the image scale. A higher ISI value indicates a sharper image, as the image intensity variation tends to be smaller when blurriness is stronger [40]. Three replicates were prepared per treatment, subjected to identical growth conditions, and imaged on day 7 at 1600 dpi. The following image processing steps were then applied to each image:

1. The skeleton of the visible root system (centre line) was traced manually by using the “Segmented Line” tool of Fiji [41].
2. Each of the (n) identified skeleton pixels were assigned a uniform probability (1/n) and then 4 pixels were randomly selected for each image to obtain unbiased estimates.
3. A square section of 640 × 640 pixels was formed around each of the 4 randomly selected pixels. The size of this area was empirically determined to sufficiently cover the root hair zone adjacent to the root. If any of the 4 image regions overlapped, then the process was repeated until there was no region overlap.
4. The ISI measure described above was then computed for each of the 4 image sections.

Steps 2–4 were implemented in R using a custom-developed script. The 4 × 3 values associated with each treatment were pooled and analysed using Dunnett’s T3 test for the comparison of mean values.

### Image analysis

**Fig. 1:**
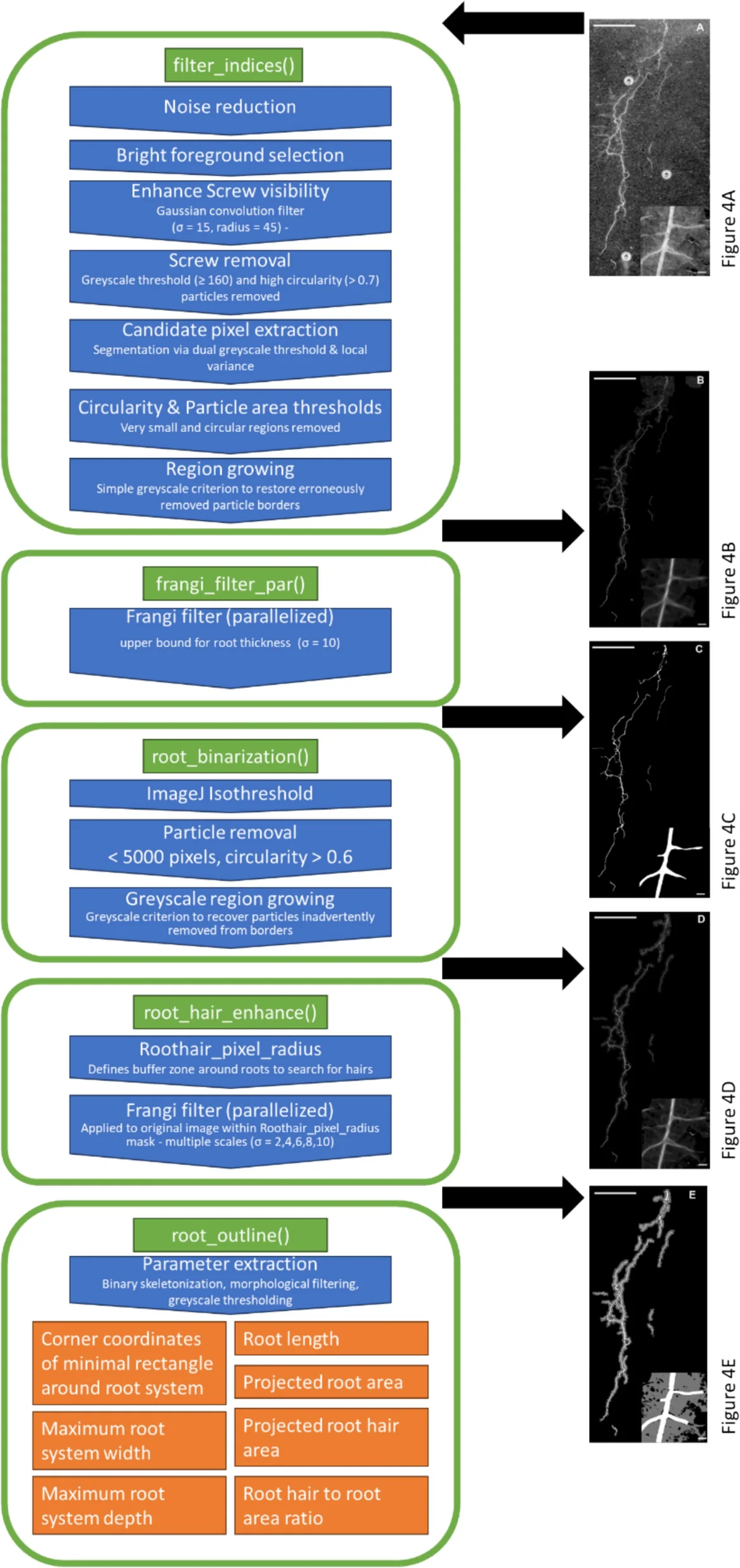
Schematic diagrams of the analysis process. Each Green ringed area represents a function created by RootHairFinder.cpp. Images generated after each function step used (see “RootHairAreaFinder.R”). Blue areas indicate processes included within each function. Orange areas indicate numerical parameters calculated. Images on the right illustrate example outputs from the analysis, showing the image matrices generated by each RootHairAreaFinder function

**Fig. 2:**
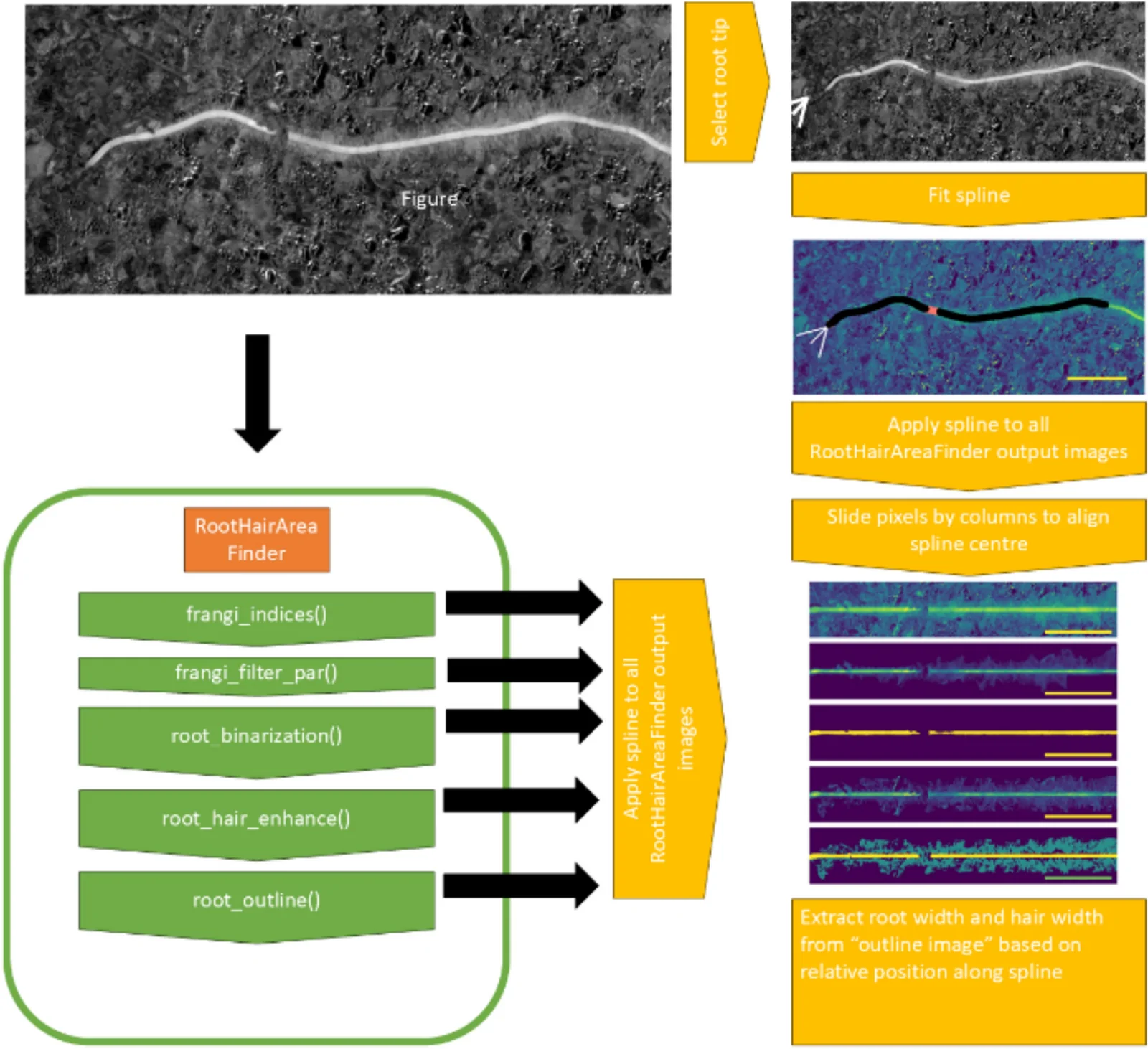
Schematic diagrams of the analysis process of a single root. Each Green ringed and green area represents a function created by RootHairFinder.cpp. Images generated after each function step used (see “RootHairAreaFinder.R”). Light orange areas indicate processes included during the single root analysis

The proposed image processing algorithm enables the analysis of root systems grown in a rhizotron unit with a black-coloured sand used as a substrate. The algorithm uses functions created by RootHairAreaFinder.cpp. An overview of the process is shown in Fig. 1, while further details of individual functions are provided in Supplementary file 3. The main outputs of the algorithm are root length, x and y dimensions of root length, total projected root area (This projected root area refers to the visible area occupied by root in the image, rather than the root’s surface area) and mean root hair width to VP root hair area width per unit segments. The individual steps which compose the algorithm are summarized in Fig. 1 (whole A3 rhizotron, Supplementary_file_4.tif) & Fig. 2 (individual root analysis, Supplementary_file_5.tif). Briefly, the algorithm uses techniques such as thresholding, mathematical morphology, particle criteria and region growth to separate foreground from background [42]. More complex filters based on Gaussian convolution procedures are also used to enhance vascular structures [43]. The final segmented image is obtained by applying the ImageJ/Fiji [] IsoData threshold [44] and region growth techniques. The VP root hair area is obtained by applying thresholds on the area adjacent to the detected roots after the process of root detection. VP root hair area is the visible projected area in the image occupied by root hairs as opposed to a measure of the surface area of the root comprised of root hairs. The algorithm is implemented in R via Rcpp [45], with dependencies on the RcppArmadillo [46] and RcppParallel [47] packages. The packages raster [48] and rgdal [49] are also used for image import, export, and visualization. Images and graphs were produced using cowplot [50], ggplot2 [51] and ggmap [52], by adding code to save each analysis function output to RDS output files. These were reloaded into R prior to visualisation. For the individual root analysis, the position of the root tip was manually selected. A spline was placed along the centre of the root binary image obtaining the midpoint of the root for each column of pixels. The x, y position of the central spline was plotted using ggplot and a rolling average of positions generated using rollmean (zoo package [53]). The new distance between each column was calculated from the diagonal distance between each column prior to aligning the columns using Pythagoras’ distance from the spline point on the x and y scales. Final images were produced by sliding vertical pixel columns to produce a linear spline. Root distance from meristem at each datapoint was calculated as pooled distances between aligned pixel columns. Using the same method each image was aligned and root hair length at each pixel columns calculated from results data.

### Root growth parameters

Root growth parameters and VP root hair area estimation were measured from the RS images obtained at 7, 14 and 21 days via analysis using the proposed algorithm described above. The variables Average Root Growth Rate (ARGR) (mm day^−1^), Average Vertical Growth Rate (AVGR) (mm day^−1^), Average Horizontal Growth Rate (AHGR) (mm day^−1^) and Average Root Hair to Root Ratio (ARHtRR) (mm mm^−1^), were used to summarise root growth and (relative) root hair abundance. Definitions for those variables are given in Supplementary file 6.

### Statistical analysis

The statistical analysis of the data was performed using the software R (version 4.4.1) and the freely available code provided by Wilcox [54]. A non-parametric 2-way ANOVA test was used to statistical compare the ranks of the experimental treatments with the function bdm2way(). Post-hoc analysis with pairwise median comparisons was performed with the mcp2a() function. Dunnett's T3 test was implemented with the function lincon(). The level of significance chosen for this study was 0.05. The graphical outputs were also produced in R with the ggplot2 [51] and the grid [36] packages.

### Image analysis validation

The projected root area output from the algorithm was validated as follows: (1) 20 rhizotron images were randomly selected among the pooled set of images for the glasshouse experiment, taken at 14 and 21 days of growth; (2) the outline of the visible root system was traced manually by using the “Polygon Selections” tool of Fiji creating regions of interest which were binarized; (3) The algorithm predicted projected root area was compared to the manual one.

Root hair detection was validated as follows: (1) 4 rhizotron images were randomly selected among the pooled set of images for the glasshouse experiment, taken at 14 and 21 days of growth; (2) 5 image sections (300 × 300 pixels) containing root hairs were subjectively chosen by a trained root scientist so that the sections spanned the length of the root system; (3) The root hair boundary was traced manually by using the “Polygon Selections” tool of Fiji; (4) The algorithm predicted root hair boundary area was compared to the manual one. In both cases, the accuracy was quantified with the Matthews Correlation Coefficient (MCC) [55, 56].

The MCC values of both traits were compared against a reference value of 0.6 using a one-sample Wilcoxon signed-rank test with a one-sided alternative hypothesis (MCC > 0.6), implemented via the wilcox_test() function from the rstatix package [57]. This threshold was selected as it is commonly interpreted as the lower bound of substantial agreement in classification and segmentation studies. Effect size (rank-biserial correlation) was computed using the wilcox_effsize() function from the same package.

Impact on the resulting VP root hair area and projected root area of varying the roothair_pixel_radius was analysed by evaluating the outpus on multiple images of varying sizes illustrating individual roots upto full rhizotron scale images. The roothair_pixel_radius was set at 30 to 250 at 20 pixel intervals. A curve was fitted to the VP root hair area vs. roothair_pixel radius using smooth.spline(, spar = 1), and the first derivative calculated using the predict(deriv = 1) function for each image tested.

### Algorithm speed

**Fig. 3:**
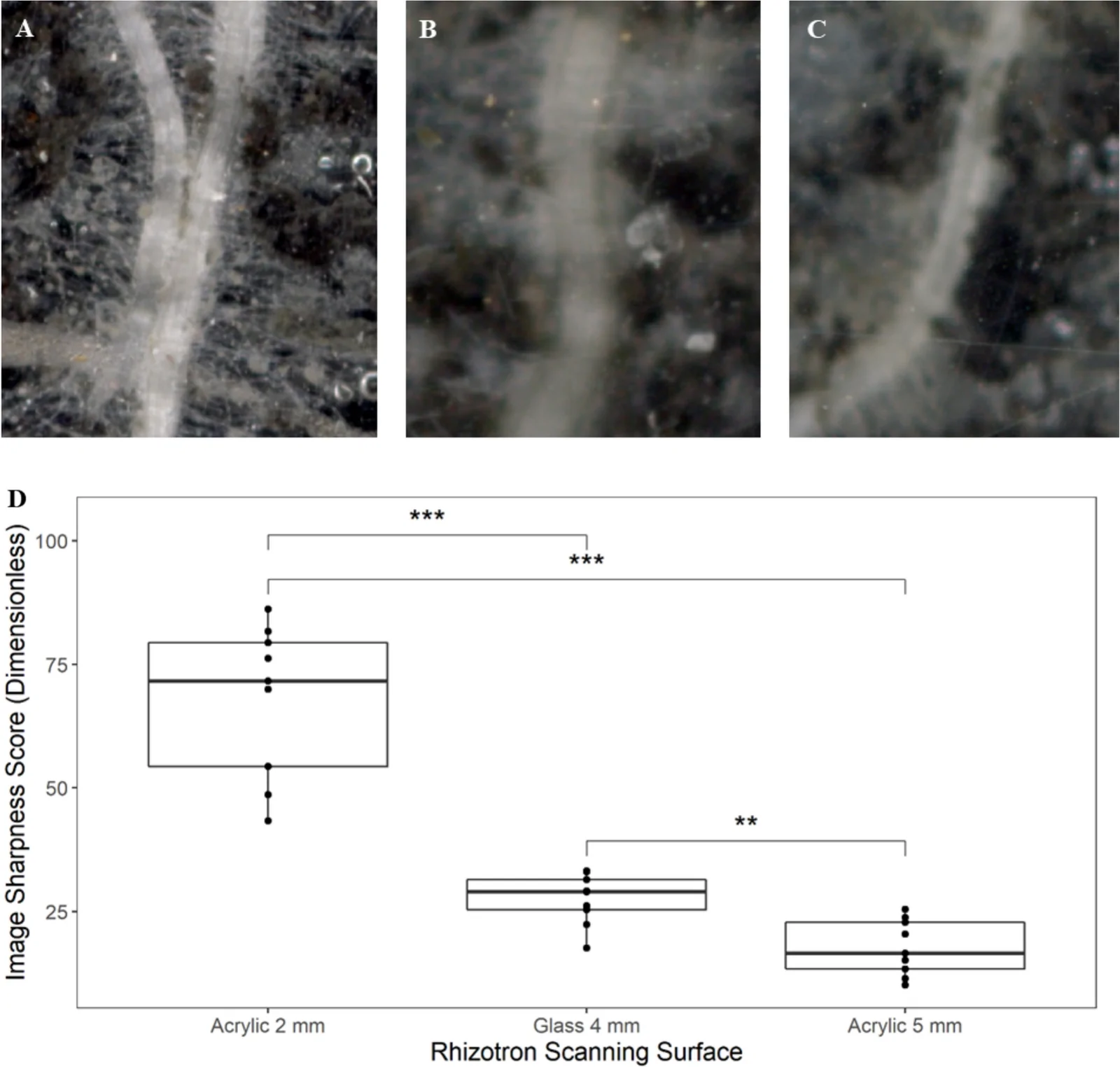
Rhizotron scanning surface experiment comparing imaging quality for **A** acrylic—2 mm thick, scanned at 1600 dpi, **B** 4 mm thick, low iron, reinforced glass, **C** acrylic—5 mm thick and. **D** ISI boxplot comparison for the three different minirhizotron scanning surfaces; A higher Image Sharpness (ISI) score indicates a higher quality image

**Fig. 4:**
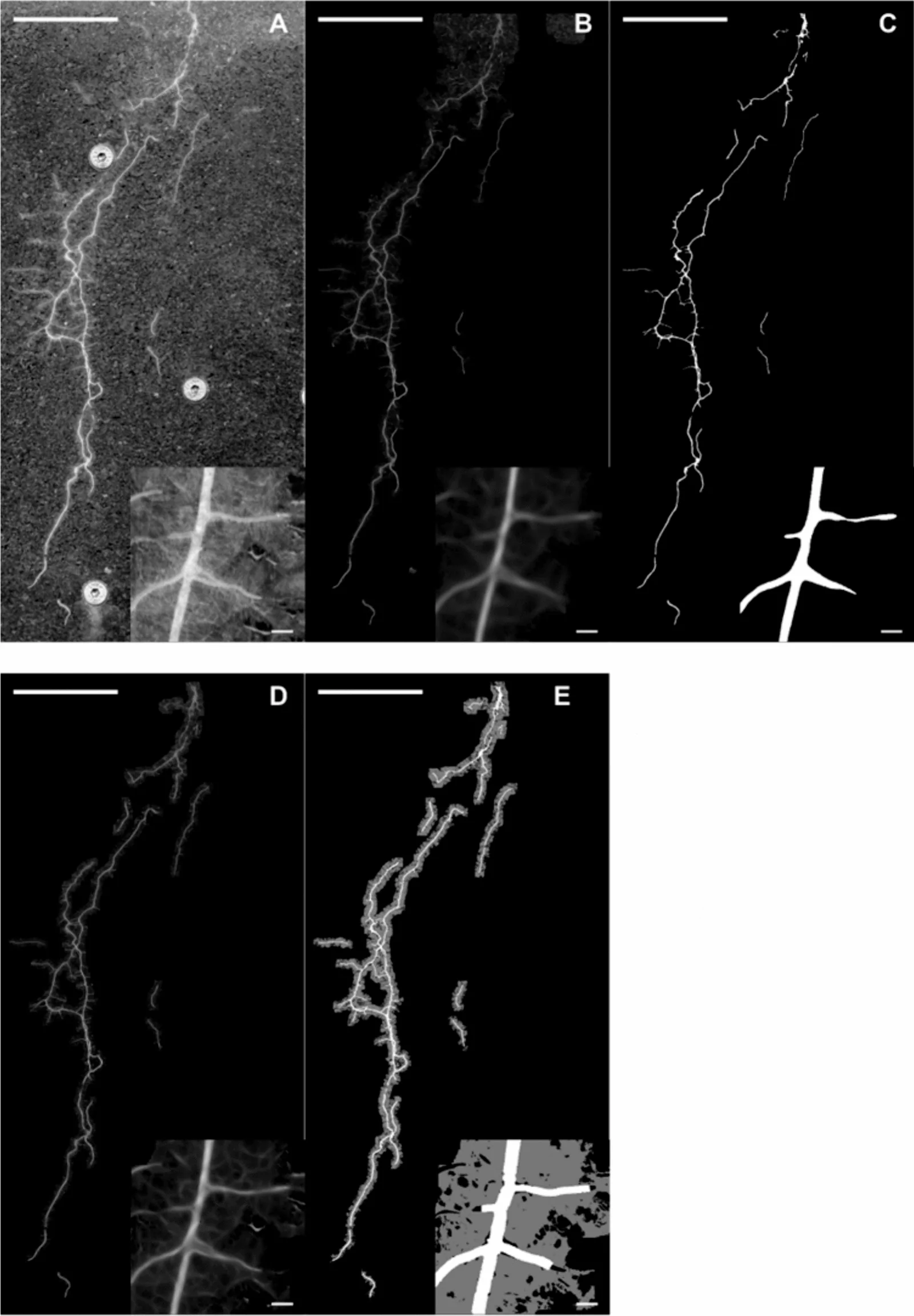
**A** Image of A3 rhizotron with 14 day old spring barley seedling obtained with using a flatbed scanner at 1200 dpi, **B** output from frangi_filter_par, **C** output from root_binarization steps, **D** output from root_hair_enhance, **E** results matrix showing roots and hair area, Main images bar = 5 cm and magnified images bar = 0.1 cm

**Fig. 5:**
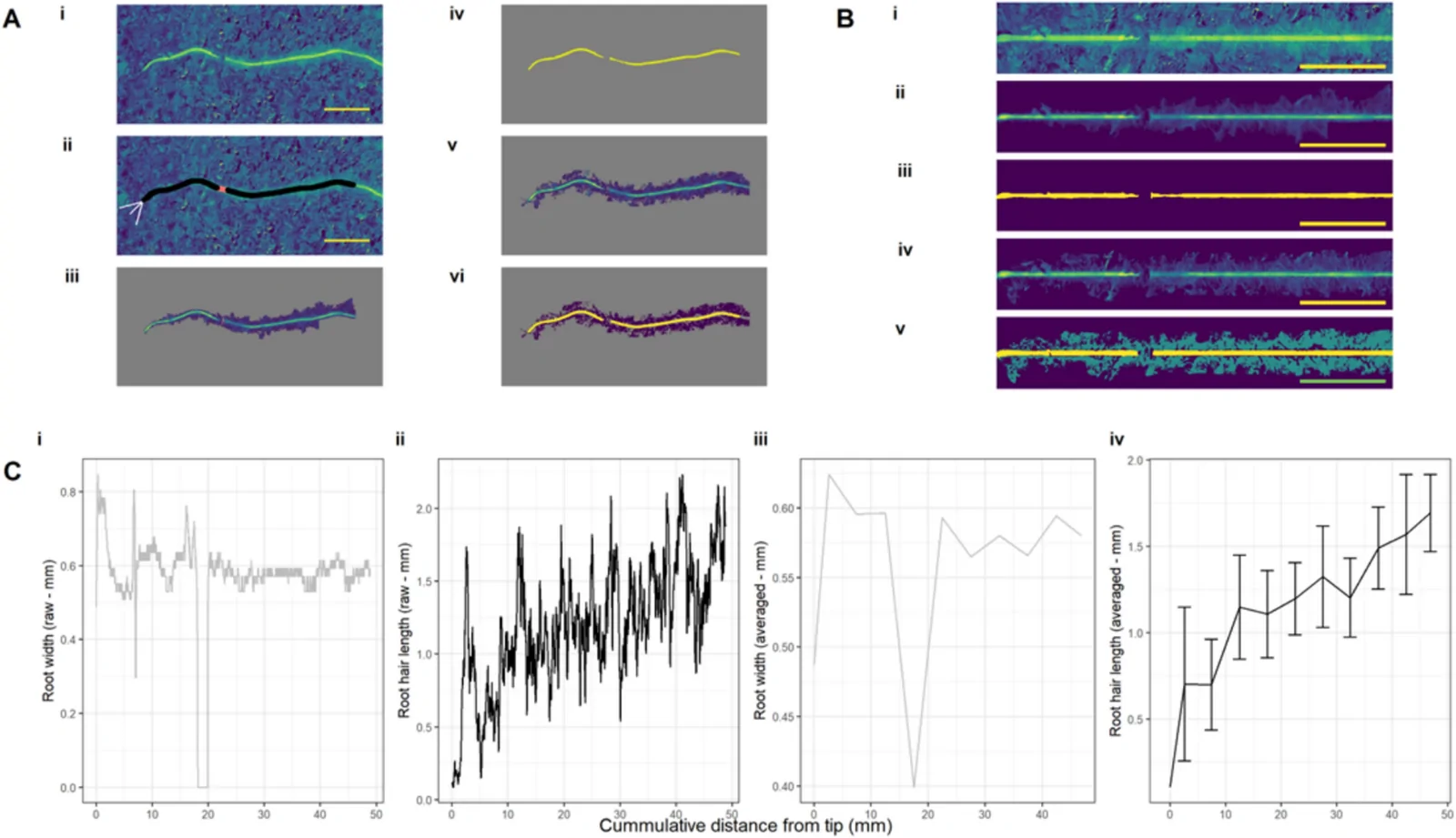
Analysis of single root: **A** Original image analysis—viridis false coloured: (i) Original image; (ii) with spline marked in black and spline fill marked in red; output from (iii) frangi_filter_par; (iv) root binarization; (v) root_hair_enhance; (vi) combined final results. **B** Extracted root output aligned along the spline (post analysis): (i) original image; (ii) frangi_filter_par; (iii) root binarization for extracting root; (iv) root_hair_enhance; (v) combined results. **C** Raw (i,ii) and averaged (iii,iv) root width (i,iii) and root hair length (ii, iv) relating to distance from the root tip. Error bars show the variance in root hair length along the 5 mm section of root ((Root hair area width—the root area width)/2). Scale bar = 1 cm

The execution speed of the algorithm was measured for; (1) a set of 8 images of 3-week-old seedlings grown in RS; (2) the images used for Figs. 4 & 5; (3) randomly cropped subsets of the image used for Fig. 4. The average (x̄ ± s) image size of the images was 106.7 ± 63.07 MB. The timings were obtained by using the R base function system.time() and extracting the user (user > elapsed, due to parallel processing) time from the outputs. Algorithm timings reported were made by using a PC with the following system properties for (1): Processor: AMD Ryzen 9 7900 12-Core Processor 3.7 GHz or for (2) & (3) Intel(R) Xeon(R) Silver 4310 CPU @ 2.10 GHz (2.10 GHz) (2 processors); Installed memory (RAM): 32.0 GB (31.2 GB usable); System type: 64-bit operating system, × 64 based processor; Windows Version: Windows 11 pro/Enterprise (respectively).

## Results

### Effect of imaging surface on image quality

The effect of different imaging surfaces on image quality is illustrated in Fig. 3. Root hairs can be seen much clearer in the image taken through the 2 mm acrylic surface (Fig. 3A), compared with the 4 mm glass and 5 mm acrylic (Fig. 3B, C respectively). These visual assessments were evaluated objectively by estimating the ISI value of each image. Figure 3D summarizes the comparison between the different scanning surfaces. The ISI scores support the visual assessments, demonstrating the sharpness of the 2 mm thick acrylic images which had a higher ISI score relative to both the 4 mm glass and the 5 mm acrylic with a 95% confidence interval of (25.033, 55.830) and (34.773, 65.668) respectively. The mean (x̄ ± s) ISI values for each treatment were 67.922 ± 15.409, 27.491 ± 5.17 and 17.702 ± 5.641 respectively.

### Assessment of image analysis performance

Root images from the glasshouse experiment (using the 2 mm acrylic imaging front) were analysed using the RootHairAreaFinder algorithm. The algorithm steps are illustrated in Fig. 1. Figure 4 shows an example image processed by the algorithm with image outputs at critical stages. An example starting image is shown in Fig. 4A, with the image after pre-processing and first Frangi filter (Fig. 4B), after root binarization (Fig. 4C), after root hair enhancement (Fig. 4D) and after root segmentation step for extracting the final results (Fig. 4E). The algorithm was estimated to have an average (x̄ ± s) execution time of 63.9 ± 41.94 s.

To assess the potential of examining root hairs along a single root and in relation to the distance from the meristem, an image with a single root was assessed using the algorithm and then post-processed (Fig. 5). Image outputs from the analysis are shown in Fig. 5A, with the user-marked root tip meristem and the calculated spline fit to the centre of the root based on the output of the RootHairAreaFinder (Fig. 5Aii). Figure 5B shows the output images from the algorithm analysis steps aligned so the spline appears linear. Using the spline to measure the distance from the root tip, the diameter of the root, and the average radius of the root hair zone was calculated. Figure 5C shows the root width (Ci, Ciii) and root hair length (Cii, Civ) at each pixel distance along the length of the root as raw data (Ci,ii) or as pooled averages of 5 mm distance from the root tip (Ciii, iv). This analysis used a roothair_pixel_radius (user selected radius of root hair zone outside of the root boundary) value of 100. Root width at distance along the root was shown to be stable except for the first 5 mm, and the section where there was a gap in the visible root system (Fig. 5Ci, iii), where root hair length along the root gradually increased with distance from the root tip (Fig. 5Cii, iv).

### Algorithm validation

**Fig. 6:**
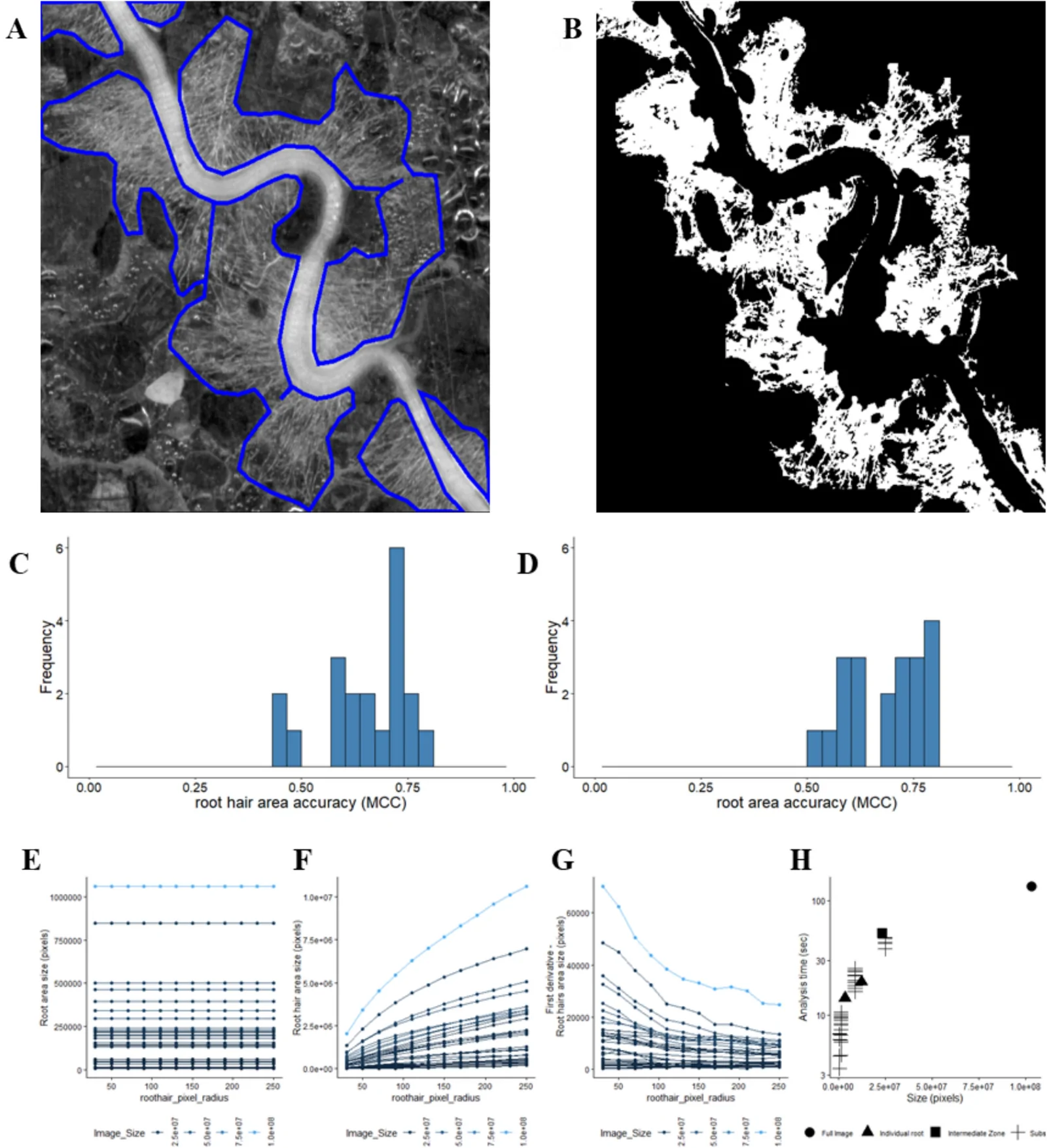
Assessment of the functioning of the root hair algorithm. Example test image showing manually traced VP root hair area (blue line) (**A**). VP root hair area (white) predicted by the algorithm after thresholding the area adjacent to the root (**B**). Histograms of Matthews correlation coefficient (MCC) for predicted VP root hair area (**C**) and projected root area (**D**), computed from pixel-wise comparisons of binarized predictions against manually traced root hair masks (300 × 300 pixels) and projected root area masks, respectively. Evaluation of the effect of increasing the roothair_pixel_radius parameter on projected root area (**E**), VP root hair area (**F**), and the first derivative of the VP root hair area (**G**). Evaluation of analysis time of images of different size ranges (**H**)

The validation procedure for the algorithm is summarized in Fig. 6. The manually traced VP root hair area enclosed by the traced root hair boundary illustrated in Fig. 6A (blue line), was compared with the algorithm predicted VP root hair area (white in Fig. 6B). Figure 6C shows the estimated MCC between the manual and automatic assessed VP root hair areas. The average (x̄ ± s) VP root hair area MCC value for the analysed sections was 0.65 ± 0.1. Similarly, Fig. 6D illustrates the estimated MCC for the projected root area detection component of the algorithm. The average (x̄ ± s) projected root area MCC value for the analysed sections was 0.68 ± 0.09. The MCC for root area was significantly greater than 0.6 (Wilcoxon signed-rank test, n = 20, p = 0.002), with a large effect size (r = 0.62). Root hair area showed a similar but weaker effect, with MCC values also significantly exceeding 0.6 (n = 20, p = 0.022) and a moderate effect size (r = 0.45).

The roothair_pixel_radius sets the area around the root that is searched for root hairs. If the parameter is set too low, over trimming of root hairs occurs producing an output underestimating the root hair region of the image. A higher value increases the potential for errors in the non hair region, and also increases the computational power required for analysis of images. To assess the effect of changing the roothair_pixel_radius on algorithm function, we assessed a range of values (from 30 to 250 in steps of 20) on the images used for Figs. 4 & 5 and cropped areas (Fig. 6E–H)). The roothair_pixel_radius did not, as expected, impact the projected root area (Fig. 6E); however, it did impact the VP root hair area depending on image size (Fig. 6F). The first derivative (Fig. 6G) of the VP roothair area against the roothair_pixel_radius shows the extent to which the area increases as the roothair_pixel_radius parameter increases. Between roothair_pixel_radius values of 100–150, the rate of increase declines for the majority of images suggesting the optimal parameter setting for this set of images is between 100–150. We also assessed the speed of the algorythm on multiple single root image (e.g. Figure 4) image, the image used in Fig. 5, and multiple subsets of that image. Figure 6H shows the relationship between the image pixel size and the speed of analysis (when not writing intermediary analysis images to drives).

### Root hair responses to environmental constraints

Table 1 shows the estimated metric potential, penetrometer resistance and air-filled porosity of each of the treatments based on the water release curves shown in supplementary file 2). The degree of saturation within the rhizotron was estimated to be 16, 27 and 36% for the 2, 4 and 6 IRE treatments respectively with the values being almost identical across DBD treatments. Based on the established water release curve of the RS substrate (Table 3), the above-mentioned values will respectively correspond to a matric potential of − 10.57, − 8.76 and − 7.66 kPa for the DBD treatment of 1.5 g cm^−3^. The mean penetrometer resistance and air-filled pore space values matching those matric suctions were respectively: 1.78, 1.68 and 1.59 MPa, and 0.41, 0.36 and 0.31%. Across the range of treatments, the lowest DBD (1.4) with the lowest irrigation, had more available water, was at the lower end of the penetration resistance scale and had more air-filled pores. Whereas the highest DBD with the highest irrigations levels, had higher moisture levels, higher penetration resistance but lower levels of air-filled pore space.

**Table 1:** Approximate matric potential (ψ), penetrometer resistance (PR) and air-filled porosity (f_a_) based on rhizotron system (RS) water release and strength curves, across dry bulk density (DBD) and irrigation event (IRE) treatments

DBD (g cm^−3^)	IRE	ψ (kPa)	PR (MPa)	f_a_ (%)
1.4	2	− 11.36	1.19	0.44
	4	− 8.99	1.12	0.38
	6	− 7.97	1.08	0.33
1.5	2	− 10.57	1.78	0.41
	4	− 8.76	1.68	0.36
	6	− 7.66	1.59	0.31
1.6	2	− 9.92	1.95	0.38
	4	− 8.45	1.85	0.33
	6	− 7.25	1.77	0.29

**Fig. 7:**
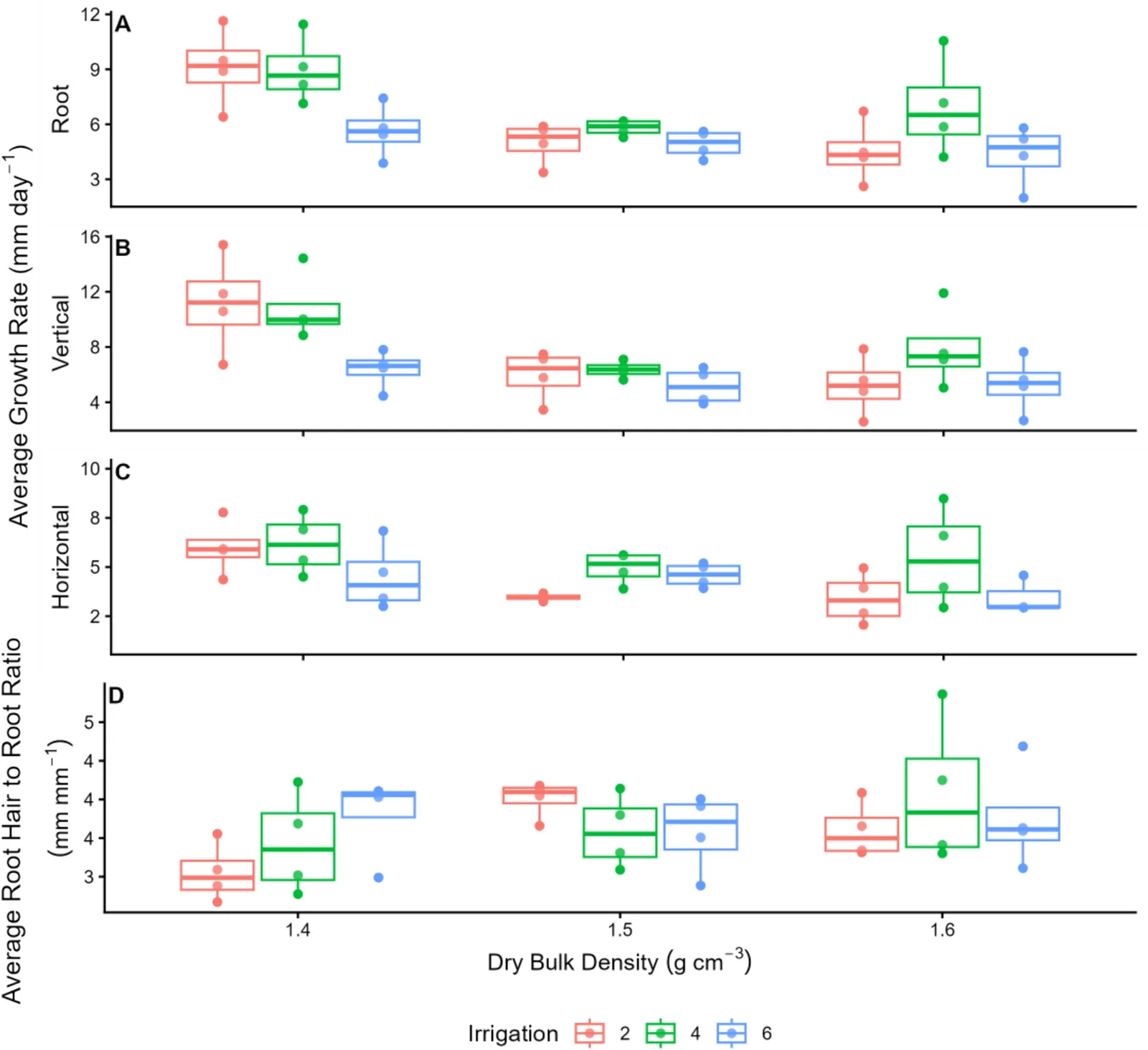
Spring barley root variables at the end of a 21-day growth period in the sand-based minirhizotron system as a function of dry bulk density (DBD) and grouped by irrigation event (IRE). Average growth rates of **A** Root (ARGR) vs. DBD, **B** Vertical (AVGR) vs. DBD and **C** Horizontal (AHGR) vs. DBD, and **D** Root Hair to Root Ratio (ARHtRR) vs. DBD

The effects of different dry bulk densities (DBD) and differential watering levels (IRE) on root growth parameters are illustrated in Fig. 7. Root responses to these conditions, average root growth rate (ARGR), average vertical growth rate (AVGR), and average horizontal growth rate (AHGR) are shown in Fig. 7A–C, respectively, while the average root hair to root ratio (ARHtRR) is presented in Fig. 7D.

For ARGR, both DBD (ANOVA F = 8.4935, p = 0.003*) and IRE (ANOVA F = 6.2317, p = 0.0086*) were significant factors, while their interaction was not (ANOVA F = 1.1427, p = 0.3627). Increased DBD reduced the AVGR across the combined IRE treatments. This was driven mainly by the difference between 1.4 & 1.5/1.6 treatments (Table 2). Increased irrigation (IRE) also decreased the ARGR across the combined DBD treatments driven mainly by the difference in growth between 2/4 IRE treatments and the 6 IRE treatment.

**Table 2:** Post Hoc comparisons for the impact on dry bulk density and irrigation treatments on root growth parameters obtained using RootHairAreaFinder

Root parameter	Comparison		psihat	P	
	DBD	IR			
ARGR	1.4: 1.5		7.2097	< 0.0001	***
	1.4: 1.6		7.876	0.0017	***
	1.5: 1.6		0.6664	0.4508	ns
		2: 4	− 2.2182	0.1319	ns
		2: 6	3.4424	0.0317	*
		4: 6	5.6606	< 0.0001	***
AVGR	1.4: 1.5		9.9035	< 0.0001	***
	1.4: 1.6		9.9211	0.003	**
	1.5: 1.6		− 0.7806	0.2654	ns
		2: 4	− 0.7806	0.2654	ns
		2: 6	5.7689	0.0100	*
		4: 6	6.5496	< 0.0001	***
AHGR	1.4: 1.5		2.851	0.0250	*
	1.4: 1.6		4.571	0.0217	*
	1.5: 1.6		1.72	0.2604	ns
		2: 4	− 3.9048	0.0284	*
		2: 6	1.20231	0.2988	ns
		4: 6	4.9278	0.0117	*

In the case of AVGR, both DBD (ANOVA F = 7.3805, p = 0.0048*) and IRE (ANOVA F = 4.4108, p = 0.0269*) were significant, with no significant interaction (ANOVA F = 0.8137, p = 0.5163). Both increased DBD and increased irrigation (IRE) reduced the AVGR across the combined IRE and DBD treatments respectively. The DBD effect was mainly driven by differences in vertical growth between treatment 1.4 and the 1.5/1.6 (Table 2), whereas the IRE effect was driven mainly by differences in 2 IR vs. 4 or 6 IRE (Table 2).

For AHGR, DBD (ANOVA F = 4.7862, p = 0.0257*) and IRE (ANOVA F = 3.9625, p = 0.0395*) were significant, but their interaction was not (ANOVA F = 1.6087, p = 0.2221). AHGR reduce with increased DBD, with differences mainly driven by differences between DBD 1.4 & DBD 1.5/1.6 (Table 2). The highest AHGR were found in the intermediate IRE measures (Table 3) with significant differences between 2 & 4, 4 * 6 but not 2 & 6 IRE (Table 2).

**Table 3:** Root system rankings for average root (ARGR), horizontal (AHGR) and vertical (AVGR) growth rates, and root hair to root ratio (ARHtRR) medians across dry bulk density (DBD) and irrigation event (IRE) treatments

DBD (g cm^−3^)	IRE	ARGR (mm day^−1^)	AHGR (mm day^−1^)	AVGR (mm day^−1^)	ARHtRR (mm mm^−1^)
1.4	2	**9.19 (1)**	5.9 (2)	**11.22 (1)**	**2.99 (9)**
	4	8.66 (2)	**6.13 (1)**	9.98 (2)	3.35 (8)
	6	5.62 (5)	4.07 (6)	6.63 (4)	4.06 (2)
1.5	2	5.33 (6)	3.47 (7)	6.46 (5)	**4.1 (1)**
	4	5.89 (4)	5.16 (4)	6.37 (6)	3.55 (6)
	6	5.04 (7)	4.62 (5)	**5.09 (9)**	3.71 (4)
1.6	2	**4.33 (9)**	3.3 (8)	5.2 (8)	3.5 (7)
	4	6.51 (3)	5.28 (3)	7.32 (3)	3.83 (3)
	6	4.75 (8)	**2.95 (9)**	5.39 (7)	3.61 (5)

Highest and lowest ranks are highlighted in bold

Finally, for ARHtRR, neither DBD (ANOVA F = 1.2022, p = 0.3196), IRE (ANOVA F = 0.1235, p = 0.8639), nor their interaction (ANOVA F = 1.8112, p = 0.17) were significant.

Table 3, contains a summary of the rank scores of ARGR, AHGR and AVGR, for each of the experimental treatments. The largest ARGR value corresponded to the 1.4 DBD and 2 IRE treatment while the smallest corresponded to the 1.5 DBD and 6 IRE treatment. The largest AHGR value corresponded to the 1.4 DBD and 2 IRE treatment while the smallest corresponded to the 1.5 DBD and 6 IRE treatment. The largest AVGR value corresponded to the 1.4 DBD and 2 IRE treatment while the smallest corresponded to the 1.6 DBD and 2 IRE treatment. The largest ARHtRR value corresponded to the 1.5 DBD and 2 IRE treatment while the smallest corresponded to the 1.4 DBD and 2 IRE treatment.

## Discussion

### Root detection

The validation analysis enabled an estimation of the error associated with the proposed algorithm for detecting and quantifing roots grown in the RS. The average (x̄ ± s) projected root area MCC value for the analysed sections was 0.68 ± 0.09 (F1 = 0.67 ± 0.1), indicating good overall performance. Scores above > 0.5 are considered to suggest strong performance [58, 59]. Although root segmentation accuracy is good for the proposed RS, it should be stated that there are ML based root detection algorithms that achieved higher root segmentation accuracy [60]: F1 = 0.7; Narisetti et al. [61]: F1 = 0.87), but require prior model training and do not provide an estimate of VP root hair area. In contrast, the algorithm by Lu et al. [35], achieved extremely high accuracy for the species tested (average F1 = 0.97), and is also able to segment root hairs with similarly high precision. However, the algorithm is again ML-based, and runs on small images (< 400 KB—based on Supplementary file) capturing only local root patches, not whole root architecture. It is also not clear, what the runtime of the algorithm is, especially when provided with much larger images. For the proposed algorithm, the average (x̄ ± s) user time required to execute the algorithm was just 63.9 ± 41.94 s for seedlings at 3 weeks of growth, highlighting its use for analysing images in near real-time.

The proposed method is therefore a fast alternative to manual annotation, but achieves agreement with expert segmentation within the expected range for human-derived labels in complex root imaging tasks. The observed MCC values (~ 0.68) indicate performance comparable to expert-level interpretation while retaining full automation. MCC values were consistently larger than 0.6, a commonly used threshold for substantial agreement in segmentation tasks, supporting robustness across samples. Thus the approach combines expert-comparable rapid extraction of roots and root hairs via a fully automated processing pipeline, eliminating user variability, and scalability to whole-root-system images, rather than exceeding manual annotation accuracy.

Although the algorithm was validated using spring barley, it is expected to work for other plant species with bright white roots. Because the RS is standardized and the algorithm requires no prior training, unlike other ML-based methods, it is expected to generalize naturally. This could be readily verified in future experimental work.

### Root hair detection

**Fig. 8:**
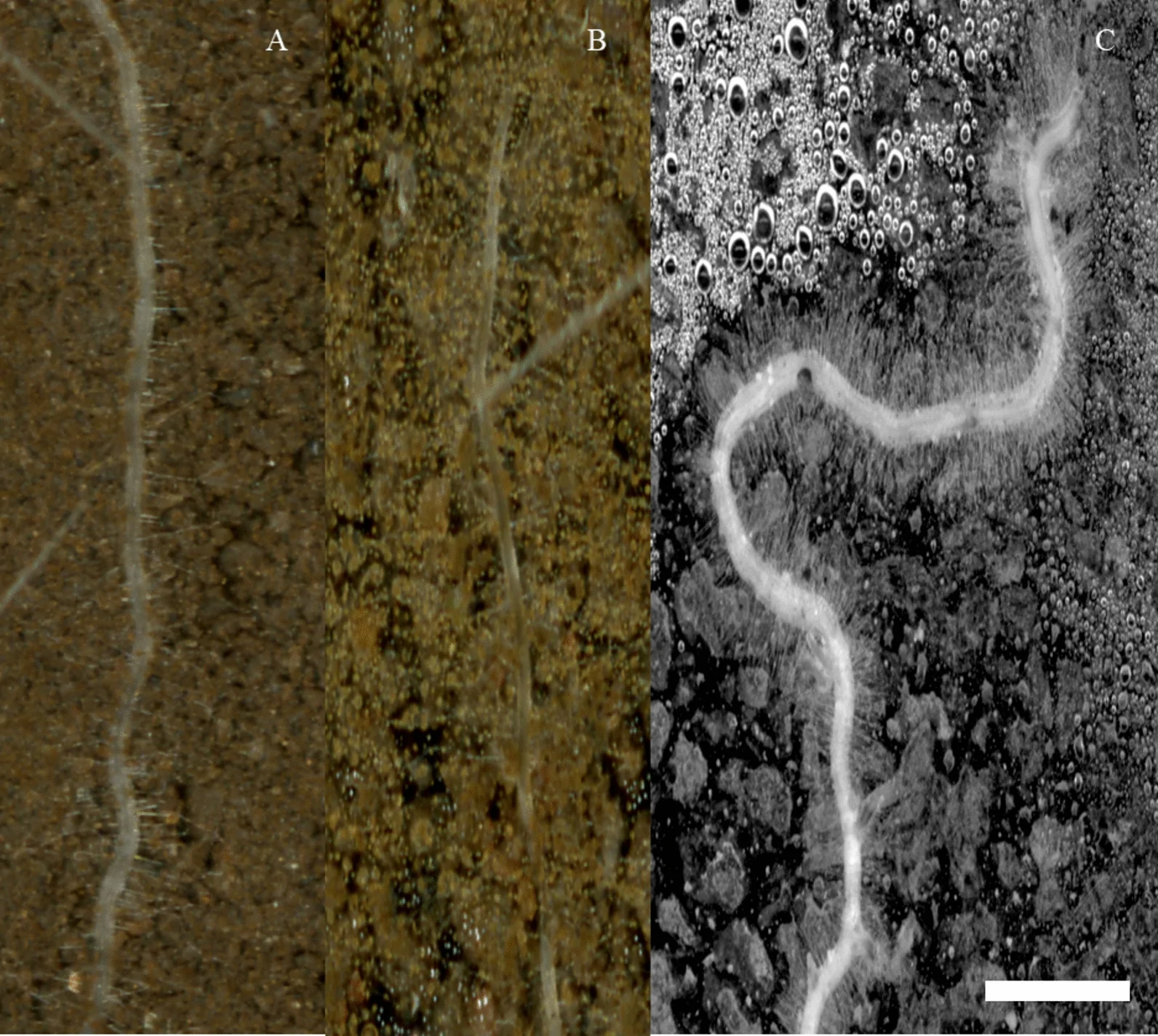
Spring barley imaged at 1600 dpi after 14 days of growth inside the rhizotron system. The swelling behaviour of both clay (**A**) and sandy (**B**) soils results in a significant fraction of the root material being covered by soil particles, especially fine structures such as root hairs. In contrast, such effects could be significantly reduced by using non-swelling inorganic sand (**C**) as a growth substrate. Bar = 4 mm

Intuitively, the ability to detect fine root structures such as hairs, will be positively correlated with image quality. As such, the first step was in assessing in a more objective way the effect of different imaging surfaces on image quality. The method used to quantify image sharpness although simple, tends to be more robust to noise relative to most other candidates [62] and led to the selection of a 2 mm thick acrylic object (Fig. 3) for scanning. The next challenge was to reduce the effect of the growth substrate itself on image quality. Figure 8, illustrates why sand was selected as a substrate as opposed to the more mechanically complex soil since its shrink-swell cycles effectively lead to particles covering most of the root material.

As for the validation, the segmentation accuracy for VP root hair area was similar to that of roots, achieving an MCC value of 0.65 ± 0.1 (F1 = 0.73 ± 0.1). The observed variability in MCC values across images reflects differences in image complexity, including variation in root density, root hair visibility, and background texture inherent to minirhizotron imaging. Despite this spread, all MCC values remained positive, with a minimum of 0.46, indicating consistent agreement between predicted and ground truth segmentation across heterogeneous conditions. Across the three cultivars used to validate their algorithm, Vicent et al. [30] obtained F1 scores ranging from 0.32–0.72, with an average value of 0.48 (estimated from Table 3 from Vicent et al. ). The accuracy of their method was generally lower than that of the proposed algorithm, but was comparable for papaya grown in peat and perlite, likely due to the dark root tones. However, the method is semi-automated, requiring user input, and focuses on relatively small regions of the root system. Conversely, similar for root segmentation, the algorithm of Lu et al. [35] achieved a higher F1 score 0.852 for VP root hair area. However, the same limitations mentioned above for root segmentation also apply for the root hair component of that algorithm. Neither of the previously mentioned algorithms can extract overall root architecture and root hairs from a single image while also maintaining locational information. However it should be noted that the analysis is restricted to the focal plane of the image which means that there will always be root hairs that are not visible, either because they are out focus or are covered by other root material or sand particles. This is however, an intrinsic limitation of the RS, where the analysis is restricted to the thin interface of the scanning surface and the substrate/root mixture. Furthermore, although a number of authors tend to consider only clearly visible root hairs [26, 63] in their analysis, this was avoided here because it was found to give a misleading estimate of the total VP root hair area, especially at areas of high root hair density. Another limitation is that the algorithm identifies VP root hair area but not actual root hair count or more complex parameters. This is unfortunately not possible with the RS as the images are restricted to a 2D image plane. Furthermore, because the root hairs were 1 pixel in width, the process of correctly counting them will be especially challenging and likely prone to errors [30, 35].

### Root growth responses to experimental treatments

This ranges of penetrometer resistance, matric potential and air-filled pore space imposed in this study fall within those commonly associated with the least limiting water range, where root growth is jointly constrained by water availability, aeration and mechanical impedance [64]. The review of Bengough et al. [7] suggested that in maize root elongation rate is halved at a matric potential of -500 kPa and a penetrometer resistance value of 2 MPa. Similarly in soils, spring barley roots have been shown to decrease root elongation rates by more than 50% in response to penetrometer resistance measures of 1 MPa at a matric potential of − 20 kPa [38]. The matric potential achieved in this study ranged from only a fraction of that magnitude was achieved in this study ranged from 10.57 to − 7.66 kPa. The reason for this narrow range in matric suction and (relatively) larger range in air-filled pore space was clearly due to the dominance of the large pores in the RS substrate and the level of irrigation. Nevertheless, the gradual decrease offered a spectrum of increasing water availability and enabled the manipulation of water stress, although it was restricted to the macro-pore scale, with an average absolute treatment difference of 20.14% in ARGR between the 2 and 6 IRE treatments for each DBD treatment. The estimated penetrometer resistance range was also from 1.08 to 1.95 MPa. This range of penetrometer resistance has been shown to halve barley root growth in soil [38] and similarly the root growth in the RS system was more than halved by the increased DBD treatments (Fig. 7). The reduction in root growth indicates that an increase in the DBD translates to an increase in mechanical impedance by reducing root elongation rates. Our findings were thus, in agreement with the general trend of an inverse relationship between DBD and root elongation rates [65].

Bengough et al. [66] concluded that root hairs enhanced root penetration ability in low density (1.0–1.2 g cm^−3^) soils but not to higher density (1.5 g cm^−3^). In this study, no significant differences could be detected across the 1.4–1.6 g cm^−3^ density range (p = 0.3196). Although, the difference in penetrometer resistance was large enough to induce a statistically significant difference (1.4 DBD treatment) in terms of ARGR this effect was perhaps not enough to induce a change in VP root hair area. Root hairs are also critical for solute absorption [67] and as such, intuitively at least, one might expect a reduced ARHtRR for the higher IRE treatments. However, there was no evidence of a statistically significant difference between the IRE treatments (p = 0.8639). Results from Haling et al. [68] also suggest a differential response in root hair length among different strength and water treatments. Although, root hair length is not reported here a differential response between treatments will have affected the estimates of VP root hair area which will in turn affect ARHtRR but, no such variation was detected here. However, it must be considered that the minimum and maximum penetrometer resistance values of the treatments used by the authors were 0.03 and 4.45 MPa respectively so the range used was wider than the range used here, i.e., 1.08 to 1.95 MPa. In a similar manner, the low and high-water concentration treatments had a matric potential of around − 7.5 and − 40 kPa respectively with a significantly lower matric potential limit in comparison to the one used in this study, i.e., − 11.36 kPa. This could help explain why the corresponding ARHtRR responses were not detected in the results reported here.

The growth material used here was a mixture of sieved gravel and sand combinations, and the combination used produced an open matrix. However, further evaluation of size fractions along with root and root hair evaluations using the algorithm presented here, including smaller particle ranges, and a wider range of cultivars will allow further understanding of the impact of soil physical conditions on root and root hair responses to soil physical conditions.

## Conclusions

This study demonstrates that the proposed RS, combined with a non-ML image analysis algorithm, provides a robust, fast, and potentially generalizable approach for simultaneous quantification of whole-root architecture and VP root hair area from single images. Validation analysis showed good segmentation performance for both roots and root hairs (MCC ≈ 0.65–0.68; F1 ≈ 0.67–0.73), comparable to or exceeding existing semi-automated approaches, while avoiding the need for prior model training. Although higher accuracies have been reported for ML-based methods, these typically operate on small image patches, require training data, and do not readily scale to whole-root systems. In contrast, the proposed method enables near real-time analysis of large images, making it well suited for high-throughput or time-sensitive experimental workflows.

Application of the method to controlled water and bulk density treatments confirmed that the RS can impose realistic gradients of matric potential, aeration, and mechanical impedance within the least limiting water range. Root growth responses were primarily driven by changes in dry bulk density, with increased mechanical impedance significantly reducing axial root growth rates, consistent with established relationships reported in the literature. In contrast, no significant treatment effects were detected for relative VP root hair area, likely reflecting the comparatively narrow ranges of matric potential and penetrometer resistance achievable in the RS compared to studies spanning more extreme conditions.

Overall, the proposed RS and analysis framework offers a practical compromise between accuracy, speed, and scalability, enabling integrated assessment of root system architecture and root hair development under controlled physical soil conditions. Future work should focus on extending validation to additional plant species, exploring wider ranges of mechanical and drought stresses, and integrating complementary imaging steps to further enhance accuracy.

## Supplementary Information

Supplementary Material 1.

Supplementary Material 2.

Supplementary Material 3.

Supplementary Material 4.

Supplementary Material 5.

Supplementary Material 6.

Supplementary Material 7.

Supplementary Material 8.

## Data Availability

The RootHairAreaFinder.cpp file, R script and example data files for rhizotron analysis will be made available through github (https://github.com/TracyValentine/RootHairAreaFinder) and zenodo (https://zenodo.org/uploads/19288922).

## Abbreviations

RSA: Root system architecture
DBD: Dry bulk density
IRE: Number of irrigation events
ISI: Image Sharpness Index
ARGR: Average Root Growth Rate
AVGR: Average Vertical Growth Rate
AHGR: Average Horizontal Growth Rate
ARHtRR: Average Root Hair to Root Ratio
MCC: Matthews Correlation Coefficient
