# Supplementary material for: RootHairAreaFinder: an image processing method for quantifying barley root growth and root hairs simultaneously in a flat rhizotron system
Source: Plant Methods. 2026 Jul 12;22:86. doi: 10.1186/s13007-026-01564-z (PMC13644045; doi:10.1186/s13007-026-01564-z)
Supplement: Supplementary file 2 [file 13007_2026_1564_MOESM2_ESM.docx]

## Supplementary file 2

The “Flourite Black Sand” came in two different size fractions both gravel (8–4 mm) and sand (4–0 mm). The gravel and the sand were first mixed in a 50:50 ratio (gravimetric) and the entire mixture was subsequently sieved at 4.0, 2.8, 2.0 and 1.4 mm to create mixtures of 4.0–2.8, 2.8–2.0, 2.0–1.4 and 1.4–0 mm respectively. Each mixture was then washed with tap water for three times and subsequently oven dried at 105 °C.

A series of cylindrically shaped (4.5 mm diameter x 5.0 mm height) repacked sand cores were packed at different dry bulk densities (1.4, 1.5 and 1.6 g cm^-3^) (n = 4). The gravimetric moisture content of each sand core was first adjusted to 20 % to give it consistency. Cores were saturated with degassed water, then subjected to a sequence of different matric suctions (5, 10, 20 and 50 kPa) *via* the use of sand and tension tables. A needle penetrometer (1 mm diameter, 30° cone angle, 4 mm min^-1^ penetration rate, readings; averaged at 1–mm intervals from 5–15 mm depth range) fitted to a mechanical test frame (Instron model 5544; Instron, MA, USA), with a 50 N load cell accurate to 2 mN at maximum load, was used to measure penetration force at each matric suction and calculate penetrometer resistance (force divided by cone cross-sectional area) (37,38) (69,70). The measurements of mass and penetrometer resistance enabled the estimation of the sand water release and strength curves respectively (Figure S2). This enabled an assessment of the differences between samples with contrasting physical properties and how that may affect their strength and water holding capacity.

**Figure S2**: Sample mean ± standard deviation of rhizotron system substrate strength curves (**A**) and water release curves (**B**) for dry bulk density values of 1.4, 1.5 and 1.6 g cm^-3^ with connecting lines for the mean values.
