# Supplementary material for: RootHairAreaFinder: an image processing method for quantifying barley root growth and root hairs simultaneously in a flat rhizotron system
Source: Plant Methods. 2026 Jul 12;22:86. doi: 10.1186/s13007-026-01564-z (PMC13644045; doi:10.1186/s13007-026-01564-z)
Supplement: Supplementary file 3 [file 13007_2026_1564_MOESM3_ESM.docx]

## Supplementary file 3

filter_indices(grey_matrix)

The first step pre-processes the image to reduce noise and select a subset with a high probability of being foreground. A Gaussian convolution filter (σ = 15, radius = 45) is applied to enhance screws visible in the rhizotron images. A grayscale threshold (≥ 160) is then applied, and particles with high circularity (> 0.7) are removed.

The remaining candidate pixels are further segmented using a dual threshold based on grayscale intensity and local variance (2 × 2 kernel). Circularity and particle area thresholds are applied to the resulting mask to isolate potential foreground pixels. A region-growing algorithm based on a simple grayscale criterion is then used to restore pixels that may have been erroneously removed along particle borders. These pixels are passed to the next step.

frangi_filter_par(grey_matrix, foreground_id, sigma)

This step enhances vascular structures by applying a parallelized version of the Frangi filter to the candidate pixels from the previous step. A hyperparameter σ value of 10 is used as the upper bound for root thickness.

root_binarization(vessels_root)

The enhanced vascular image is thresholded to extract the root foreground. The ImageJ IsoData threshold is first applied, after which particles are removed based on size (< 5000) and circularity (> 0.6). A region-growing algorithm, based on a grayscale criterion, is then used to recover pixels that may have been mistakenly removed along the borders of remaining particles.

root_hair_enhance(grey_matrix, root_binary, roothair_pixel_radius)

The resulting binary root image is analyzed to enhance root hairs extending beyond the detected roots. The hyperparameter roothair_pixel_radius defines a buffer zone around the roots within which root hairs may be present. The Frangi filter is applied again to the original grayscale image at multiple scales (σ = 2, 4, 6, 8, 10), and the maximum response across scales is retained.

root_outline:

The final step uses information from the previous stages to quantify root traits such as root length and projected areas of roots and root hairs. These traits are extracted using a combination of binary skeletonization, morphological filtering, and grayscale thresholding.
