# Supplementary material for: RootHairAreaFinder: an image processing method for quantifying barley root growth and root hairs simultaneously in a flat rhizotron system
Source: Plant Methods. 2026 Jul 12;22:86. doi: 10.1186/s13007-026-01564-z (PMC13644045; doi:10.1186/s13007-026-01564-z)
Supplement: Supplementary file 4 [file 13007_2026_1564_MOESM6_ESM.docx]

## Supplementary file 6

The following definitions are used for the variables that characterise root growth in images of the rhizotron system (RS):

$$F1=\frac{2*TP}{2*TP+FP+FN}$$

$$MCC=\frac{\left( TP*TN \right)-(FP*FN)}{\sqrt{\left( TP+FP \right)*\left( TP+FN \right)*\left( TN+FP \right)*(TN+FN)}}$$

where TP, TN, FP, and FN correspond to true positive, true negative, false positive, and false negative pixels obtained by comparing automated segmentation outputs against manually annotated binary masks.

- Vertical Root Length (VRL): The difference between the minimum and maximum vertical coordinates of the visible root system grown in the RS. The measured value is expressed in units of mm.
- Horizontal Root Length (HRL): The difference between the minimum and maximum horizontal coordinates of the visible root system grown in the RS. The measured value is expressed in units of mm.
- Vertical Growth Rate (VGR): The difference between the VRL values at 2 different time points (t) divided by the time interval (δt), i.e.,
  - ${VGR}_{ti}= {[(VRL}_{ti}- {VRL}_{t\left( i-1 \right)}) / \delta t]$.
  - The calculated value is expressed in units of mm day^-1^.
- Horizontal Growth Rate (HGR): The difference between the HRL values at 2 different time points (t) divided by the time interval (δt), i.e.,
  - ${{HGR}_{ti}=[(HRL}_{ti}- {HRL}_{t\left( i-1 \right)}) / \delta t]$.
  - The calculated value is expressed in units of mm day^-1^.
- Root Growth Rate (RGR): The weighted sum of the VGR and HGR values. The weights are defined by the corresponding RS dimensions of height (400 mm) and length (261 mm), i.e.
  - ${R{GR}_{ti}=(VGR}_{ti}* 0.605144)+ {(HGR}_{ti}* 0.394856)$
  - The calculated value is expressed in units of mm day^-1^.
- Average Root Growth Rate (ARGR): The average value of the RGR values from each time point (t), i.e.,
  - $ARGR= {(\sum_{ti=1}^{ti= n} {RGR}_{ti})}/n$
  - The calculated value is expressed in units of mm day^-1^.
- Average Vertical Growth Rate (AVGR): The average value of the VGR values from each time point (t), i.e.,
  - $AVGR= {(\sum_{ti=1}^{ti= n} {VGR}_{ti})}/n$
  - The calculated value is expressed in units of mm day^-1^.
- Average Horizontal Growth Rate (AHGR): The average value of the HGR values from each time point (t), i.e.,
  - $AHGR= {(\sum_{ti=1}^{ti= n} {HGR}_{ti})}/n$
  - The calculated value is expressed in units of mm day^-1^.
- Root Hair to Root Ratio (RHtRR): The ratio of the VP root hair area (VPRHA) to the projected root area (PRA).
  - $RhtRR=VPRHA/PRA$
  - The measured value is dimensionless (mm mm^-1^).
- Average Root Hair to Root Ratio (ARHtRR): The average value of the RHtRR values from each time point (t), i.e.,
  - $ARHtRR = {(\sum_{ti=1}^{ti= n} {RHtRR}_{ti})}/n$
  - The calculated value is dimensionless.
